# Contribution of Foods and Poor Food-Handling Practices to the Burden of Foodborne Infectious Diseases in France

**DOI:** 10.3390/foods9111644

**Published:** 2020-11-11

**Authors:** Jean-Christophe Augustin, Pauline Kooh, Thomas Bayeux, Laurent Guillier, Thierry Meyer, Nathalie Jourdan-Da Silva, Isabelle Villena, Moez Sanaa, Olivier Cerf, on Behalf of the Anses Working Group on Consumer Information on Foodborne Biological Risks

**Affiliations:** 1 National Veterinary School of Alfort, 94700 Maisons-Alfort, France; jean-christophe.augustin@vet-alfort.fr (J.-C.A.); olivier.cerf@gmail.com (O.C.); 2 French Agency for Food, Environmental and Occupational Health & Safety (Anses), 94700 Maisons-Alfort, France; thomas.bayeux@anses.fr (T.B.); laurent.guillier@anses.fr (L.G.); Moez.SANAA@anses.fr (M.S.); 3 Department of Psychology, Parisian Laboratory of Social Psychology (LAPPS), University Paris Nanterre, 92000 Nanterre, France; thierry-marcel.meyer@parisnanterre.fr; 4 French National Public Health Agency, 94415 Saint-Maurice, France; nathalie.jourdan-dasilva@santepubliquefrance.fr; 5 Laboratory of Parasitology—Mycology, EA ESCAPE, University of Reims Champagne-Ardenne, 51100 Reims, France; ivillena@chu-reims.fr

**Keywords:** foodborne disease burden, food safety, foodborne pathogens, food-handling practices

## Abstract

The foodborne disease burden (FBDB) related to 26 major biological hazards in France was attributed to foods and poor food-handling practices at the final food preparation step, in order to develop effective intervention strategies, especially food safety campaigns. *Campylobacter* spp. and non-typhoidal *Salmonella* accounted for more than 60% of the FBDB. Approximately 30% of the FBDB were attributed to 11 other hazards including bacteria, viruses and parasites. Meats were estimated as the main contributing food category causing (50–69%) (CI90) of the FBDB with (33–44%), (9–21%), (4–20%) (CI90) of the FBDB for poultry, pork and beef, respectively. Dairy products, eggs, raw produce and complex foods caused each approximately (5–20%) (CI90) of the FBDB. When foods are contaminated before the final preparation step, we estimated that inadequate cooking, cross-contamination and inadequate storage contribute for (19–49%), (7–34%) and (9–23%) (CI90) of the FBDB, respectively; (15–33%) (CI90) of the FBDB were attributed to the initial contamination of ready-to-eat foods—without any contribution from final food handlers. The thorough implementation of good hygienic practices (GHPs) at the final food preparation step could potentially reduce the FBDB by (67–85%) (CI90) (mainly with the prevention of cross-contamination and adequate cooking and storage).

## 1. Introduction

The burden of foodborne illnesses (i.e., estimates of the annual numbers of foodborne illnesses and associated hospitalizations and deaths) was estimated in France for 15 major pathogens [1]. These pathogens were estimated to account for 1.5 million cases of foodborne illnesses (90% credible interval, CI90, 1.3–2.2 million) each year in France. Among these foodborne pathogens, *Campylobacter* spp., non-typhoidal *Salmonella* and norovirus were responsible for 73% of all illnesses. These estimates are of primary importance to set priorities for surveillance, prevention and control strategies [1].

Regarding the prevention and intervention options, it is also essential to attribute the disease burden to food commodities and food-handling practices. Analysis of foodborne outbreaks investigations has been shown useful to attribute disease caused by several pathogens [2,3,4,5]. However, even though the notification of foodborne outbreaks is mandatory in France, these notified cases constitute a small part of the total number including sporadic cases. For instance, 119 outbreaks of salmonellosis were notified in France in 2016 [6] accounting for 1047 cases of illnesses among the total estimated number of 183,000 cases (CrI90 102,000–388,000) corrected for underreporting and including sporadic cases. Moreover, the proportion of outbreaks for which the transmission route is known with strong evidence is low, strong-evidence outbreaks in the European Union (EU) accounted in 2018 for 709 13.8% of all reported outbreaks [7]. As a result, the proportion of salmonellosis cases for which the transmission route is known with strong evidence is very low. Source attribution studies using microbiological subtyping were performed in France for *Salmonella* [7] and *Campylobacter* spp. [8,9]. These studies are interesting with which to assess the relative contribution of different food-animal reservoirs and to justify control measures at the primary production but are unable to identify implicated food vehicles and food-handling practices.

Some foodborne illnesses are associated with poor handling practices at the final preparation step (in household or food service establishments) such as improper storage, inadequate cooking, or cross-contamination [10]. The importance of the role of consumers is also confirmed by data from foodborne outbreak investigations in France. About one-third of foodborne outbreaks reported in France occur within the family (between 26% to 39% depending on the year) [6]. Thus, food safety information aimed at consumers could help reduce the foodborne disease burden. Likewise, training programs are often mandatory for professional food handlers [11].

The major measures to control pathogens such as cleaning and disinfection of premises, equipment and hands, or appropriate storage and cooking temperature are well known [12,13] but the attribution of the foodborne disease burden (FBDB) to the poor food-handling practices needs to be quantified in order to develop targeted prevention campaigns.

The objective of this study was to identify key foods and food-handling practices associated with each foodborne pathogen disease in France in order to develop effective intervention strategies. This approach will attribute the FBDB among foods and food-handling practices at the preparation step at home and in food service establishments.

## 2. Materials and Methods

### 2.1. Working Group

A working group was constituted by the French Agency for Food, Environmental and Occupational Health and Safety (ANSES). Attention was paid to avoiding conflict of interests and to ensuring that the expertise of the 15 expert members adequately reflected the aims of the study: foodborne diseases (epidemiology, infectious diseases, risk assessment, food science, bacteriology, virology, parasitology) and social sciences (sociology, public policy, communication sciences, social psychology). The collective expertise was conducted on the basis of French epidemiological data to estimate the burden of foodborne diseases and on a literature search on the risk factors (foods and practices) of foodborne diseases. The literature data used to complete the source attribution are presented in Appendix A.

### 2.2. Estimating the Burden of Foodborne Diseases

The FBDB in France was assessed by combining the incidence of major foodborne pathogens and the severity of the resultant diseases. The 26 major foodborne pathogens or hazards (including histamine, and differentiating the congenital and acquired forms of toxoplasmosis) considered in the study are presented in Table 1 and Table 2. An approach based on scoring was applied to characterize the incidence and the severity of diseases. The use of broad categories for the scoring indirectly takes into account data uncertainties [14] for incidence and severity.

The incidence of foodborne diseases was taken from a study performed in France for the period 2008–2013 [1,15] for 15 pathogens among the 26 hazards or was estimated from surveillance data collected by the National Public Health Agency (*Santé Publique France*) and by National Reference Centers (NRCs). The estimated annual incidence of diseases per 100,000 persons in France was scored with a decimal logarithm scale as follows: score = 0, <0.01 cases; score = 1, 0.01–0.1 cases; score = 2, 0.1–1 cases; score = 3, 1–10 cases, score = 4, 10–100 cases, and score =5, >100 cases.

The severity of diseases was expressed as the disability-adjusted life year (DALY) per case which summarizes the impact of morbidity and mortality of diseases in a single measure [16]. The DALYs, expressing then the numbers of years lost due to disability or early death, were also categorized using a decimal logarithm scale as follows: score = 1, <10; score =2, 10–99; score = 3, 100–999; and score = 4, ≥1000 per 1000 cases of illnesses. Published studies [17,18,19] were primarily used in deriving the score of the DALYs for diseases. Expert opinion of epidemiologists and experts in infectious diseases was subsequently used when no estimate was published for some diseases or when experts estimated that published DALYs were not relevant. In these cases, the attributed scores were derived from those of diseases characterized by similar symptoms.

The disease burden was expressed by adding incidence and severity scores (which is equivalent to the multiplication of raw estimates for incidence and severity on a linear scale). The FBDB expresses then a public health metric equivalent to DALYs. The percentage of burden attributable to each hazard was then estimated as the fraction of the exponent with base 10 of each hazard score among the total of exponents of scores.

### 2.3. Attribution of Foodborne Disease Burden to Foods and Practices

For each foodborne pathogen, the main foods considered as significant exposure routes were identified based on literature data and by expert opinion from the working group (Appendix A). Food mishandling practices that could lead to foodborne diseases were also identified for each hazard–food combination. These practices contribute to the manifestation of foodborne diseases when handling initially contaminated foods. The contributing factors were classified as (i) cross contamination, (ii) inadequate washing and disinfection of produce, (iii) inadequate processing during domestic preparation (acidification, water activity, fermentation) or cooling, (iv) inadequate freezing (insufficient temperature and/or freezing duration to destroy parasites potentially present in foods), (v) inadequate cooking (including reheating), (vi) inadequate storage (temperature and/or shelf-life).

To estimate the relative contribution of each food and food categories to the disease burden in France, the burden of each hazard was attributed to related foods by taking into account the uncertainty in their significance for the transmission of the hazard (i.e., the burden is divided by the number of foods implicated). This approach was used because no quantitative data are available to justify a more accurate source attribution process for numerous foodborne hazards or, when available, quantitative estimates are characterized by a great uncertainty. For instance, salmonellosis outbreaks in France are linked to eggs and raw egg products in approximately 50% of cases, pork meats are implicated in 15% of cases, raw milk cheeses are implicated in 10% of cases, poultry meats are implicated in less than 10% and beef meats are responsible for 5% of outbreaks [20]. This repartition can be thoroughly modified when looking at sporadic cases instead of outbreaks. David et al. [7] estimated that sporadic cases occurring in France in 2005 were also related to eggs in 50% of cases, but pork meats accounted for approximately 25% of cases, as are poultry meats, which is higher than estimates obtained with outbreaks. On the contrary, the cattle reservoir was implicated in less than 1% of sporadic cases. In another study, attributing sporadic salmonellosis to sources, the primary role of pigs and poultry was confirmed but the layers were estimated to account for only 7% of cases in France [21]. We then estimated uncertainty intervals for the relative contribution of the main identified food by performing Monte-Carlo simulations (10,000 runs) with Microsoft Excel (Microsoft Corporation, Redmond, DC, USA). Uniform distributions were used for the relative contributions of each food on the burden with minimum contributions of 5% and maximum contributions equal to (100 − (*n* − 1) × 5)%, where *n* is the number of implicated foods. These simulations allowed the estimation of 90% uncertainty intervals (5th and 95th percentiles, CI90) for the contribution for each food.

Due to the lack of quantitative data, the relative impact of poor food-handling practices on the disease burden was also assumed to be the same, i.e., uncertain range for the impact of contributing practices, for each hazard–food combination. Monte-Carlo simulations (10,000 runs) with Microsoft Excel (Microsoft Corporation) were performed using uniform distributions with a minimum relative contribution of 5% for each food-handling error to estimate 90% uncertainty intervals of the impact of contributing practices.

## 3. Results

### 3.1. Estimation of the Foodborne Disease Burden in France

The incidence and severity estimates of foodborne disease are shown in Table 1 and Table 2 respectively. The first group of hazards (*Campylobacter* spp. and *Salmonella*) accounts for 64% of the FBDB. The second group, including 11 pathogens: toxin-producing bacteria (*Bacillus cereus*, *Clostridium perfringens*, *Staphylococcus aureus*), Shiga toxin-producing *Escherichia coli* (STEC), *Listeria monocytogenes*, *Yersinia enterocolitica*, foodborne viruses (norovirus, hepatitis A and E viruses), and *Toxoplasma gondii* (congenital and acquired infections), accounts for a 35% of the FBDB. The remaining 13 hazards, including 8 parasites, account for 1% of the FBDB.

### 3.2. Attribution of Disease Burden to Foods and Practices

The major exposure routes and food-handling practices were identified for each foodborne hazard (Table 3). Appendix A gives the details of the data used by the working group to justify the expert opinion about source attribution for each foodborne hazard. These are the foods most frequently contaminated or responsible for the majority of outbreaks.

**Figure 1:**
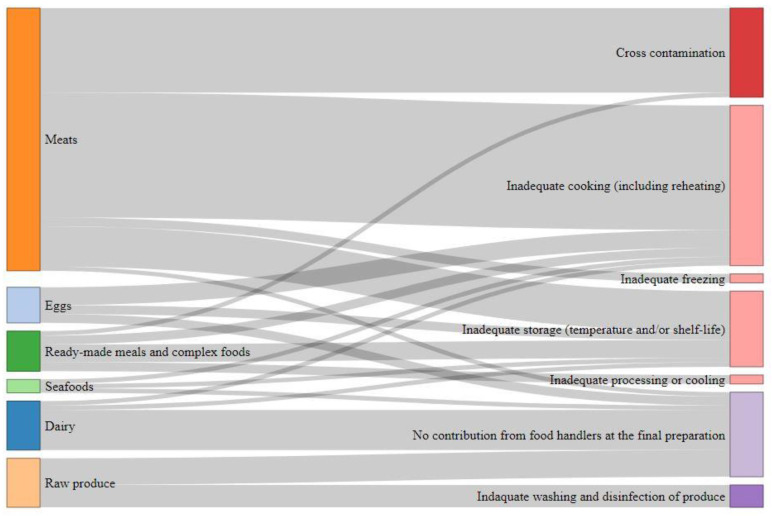
Sankey diagram of the foodborne disease burden according to the different food categories and the consumers practices (detailed percentages are given in Table 5).

Figure 1 illustrates the burden distribution by food category and consumer practices. Meats are estimated as the main contributing food category causing (50–69%) (CI90) of the FBDB (Table 4). Among them, poultry meat is the main contributor ((33–44%) (CI90) of the FBDB). Dairy products, eggs, raw produce and complex foods are estimated to cause each approximately (5–20%) (CI90) of the FBDB. Seafood are estimated as minor category with (1–6%) (CI90) of the FBDB. Raw foods (e.g., ground meat, raw milk, raw eggs products, raw fish and shellfish, etc.) account for (23–41%) (CI90) of the FBDB.

Inadequate cooking and cross contamination account for (19–49%) (CI90) and (7–34%) (CI90) of the FBDB, respectively (Table 5). These poor handling practices are mainly related to meats (Figure 1). Inadequate storage of various foods (Figure 1) is responsible for (9–23%) (CI90) of the FBDB. Inadequate washing and disinfection of produce accounts for (2–13%) (CI90) of the FBDB, and error in food processing and inadequate freezing have minor impacts. Some foodborne illness cases are linked to the consumption of ready-to-eat foods that are initially contaminated without any contributing practice of food handlers at the preparation step. These situations account for (15–33%) (CI90) of the disease burden. This is for instance the case of raw shellfish contaminated by norovirus or raw ground beef contaminated by STEC or *Salmonella*.

The thorough implementation of good hygienic practices (GHPs) at the final preparation step could reduce by (67–85%) (CI90) the FBDB (Table 5) essentially by avoiding cross-contamination, and with a correct storage and cooking which account for (60–78%) (CI90) of the burden.

## 4. Discussion

The foodborne disease burden (FBDB) related to 26 biological hazards in France was estimated and attributed to foods and poor food-handling practices at the final food preparation step. We estimated that approximately 60% of the FBDB corresponding in this study to DALYs was attributed to *Campylobacter* spp. and non-typhoidal *Salmonella*. The importance of these two pathogens was also observed by Hoffman et al. [28] who estimated that approximately 50% of the quality-adjusted life year (QALY) loss in the USA was caused by these two bacteria. Kirk et al. [18] also estimated that salmonellosis and campylobacteriosis account for approximately 50% of the DALYs for the European region. The incidence estimates used in this study are mainly derived from a French study conducted by the National Public Health Agency [1]. For most pathogens the estimated number of cases per 100,000 persons are similar to other estimates obtained in the Netherlands and USA [17,29]. Nevertheless, differences can be noticed. For instance, the incidence of cryptosporidiosis, cyclosporiasis and giardiasis, that were estimated from surveillance data from National Reference Centers and laboratory surveillance networks (Table 1), are particularly low in France. The incidences are approximately 100 lower than those published for other developed countries [18,29,30]. French estimates were based on notified cases and the actual incidence for these protozoan infections was probably underestimated but no French study was available to estimate underdiagnosis and underreporting factors. It is worth noting that, because scores based on a log_10_ scale ranging from 1 to 5 were used, even though some uncertainty is associated with incidence estimates, the scores would generally remain the same whatever the country under consideration.

The estimated DALYs published by Havelaar et al. [17] for the Dutch population in 2009 and by the Foodborne Disease Burden Epidemiology Reference Group (FERG) established by the World Health Organization (WHO) for the Europe A zone [18,19] were used as no French study estimating the severity of foodborne diseases was available. Nevertheless, the severity of toxoplasmosis published by Havelaar et al. [17] and estimated at 3170 DALYs per 1000 cases was considered as over-estimated by the experts of the working group given the very low rate of severe forms of congenital toxoplasmosis in France [31]. A score of 2 (between 10 and 99 DALYs per 1000 cases) was proposed in accordance with the estimate of Kirk et al. [18] of 60 DALYs per 1000 cases (Table 2). Based on observed clinical manifestations in France, the severity of fascioliasis provided by Torgerson et al. [19] estimated at 9000 DALYs per 1000 cases was also considered as overestimated and the working group proposed rather a score of 2 (Table 2).

In our study, meats are estimated as the main contributing food category which is in accordance with the estimates of Batz et al. [32] where meats were responsible for nearly 60% of the QALY loss in the USA. More specifically, poultry, pork and beef were responsible for 24%, 13% and 10% of the QALY loss, respectively [32]. In France, comparable estimates of (34–44%) (CI90), (9–21%) (CI90) and (4–20%) (CI90) of the foodborne disease burden were attributed to poultry, pork and beef meats, respectively. The importance of dairy products is similar in France ((5–22%) (CI90) of the burden) and in the USA (9% of the QALY loss, [32]). Raw produce and complex foods, with (6–20%) (CI90) and (8–12%) (CI90) of the burden in France, respectively, are also similar to the USA where they accounted for 10% and 12% of the QALY loss, respectively [32]. Seafood and eggs were regarded as minor sources in both countries, representing less than 10% of the disease burden or the QALY loss [32]. Finally, we can also emphasize the importance of raw foods in France that accounted for (23–41%) (CI90) of our estimate of the disease burden.

Expert opinion was used to identify the main exposure routes leading to foodborne illness because “hard” data were not available. The estimates are then sensitive to the subjective judgment of experts and results from other food source attribution studies are not fully comparable because of differences in geographic coverage, methods and food categorization [33]. For example, our study kept poultry meat as the sole main source of campylobacteriosis (Table 3) while Hoffmann et al. [33] identified also beef, dairy, and pork as sources in the EUR A subregion. However, these sources were less significant and were estimated to account for 15% to 20% of foodborne campylobacteriosis. For salmonellosis, the illnesses in the EUR A subregion are mainly attributed to eggs, pork and poultry meats by Hoffmann et al. [33] while our study allocated uniformly the salmonella FBDB to a greater list of nine food products (Table 3). However, uncertainty bounds were constructed around the estimates to take into account the uncertainty about the relative importance of each food source. The attribution of foodborne disease to foods was then estimated using uniform distributions for the relative importance of each food considered as relevant for the different hazards and contributing to at least 5% of the burden related to each hazard. Although the uncertainty intervals can be large for a specific pathogen–food combination, the uncertainty intervals for food categories derived from these distributions are relatively narrow (Table 4). For example, the burden estimate for cooked and raw ground beef is (2–13%) (CI90) while the estimate for beef is (4–20%) (CI90), and for the whole meat category is (50–69%) (CI90) (Table 4). These results show that the relative contribution of large food categories is not much affected by the uncertainty about the specific food sources even if the uncertainty bounds of these are broader. Although the uncertainty bounds are wider for food-handling practices, the phenomenon is quite similar (Table 5). Inadequate cooking, which is the main contributing factor accounts for (19–49%) (CI90) of the FBDB.

Quantitative microbial risk assessment studies often addressed the impact of some particular consumer practices on reducing foodborne risk e.g., effect of hygiene practices of consumers during the preparation of chicken meals on the salmonellosis and campylobacteriosis risks [34,35] or effect of domestic refrigerator temperature on the listeriosis risk [36] or exposure to high levels of *B. cereus* [37]. Other studies quantified the effect of specific hygiene practices on the microbial load of handled foods, e.g., effect of hygiene measures applied to cutting board, cutlery, and hands on the microbial transfer from meat to salad [38]. To our knowledge, our study is the first considering the overall attribution of the poor handling practices to FBDB. According to our study, cross-contamination, inadequate cooking and storage are responsible for (60–78%) (CI90) of the FBDB. These practices were also identified by previous studies (e.g., Medeiros et al. [12] and Taché and Carpentier [13]) but their relative contributions to the FBDB were not assessed.

Food safety prevention appears to be a domain where behavioral change could effectively reduce the FBDB. The potential reduction in disease burden when correctly applying good hygienic practices (GHPs) at the final preparation step is, however, difficult to estimate. It depends on the efficiency of control measures to limit the transfer of microorganisms, to inactivate them or to inhibit their multiplication as well as on the inclination of food handlers to apply GHPs correctly. We can assume that the prevention of cross-contamination and adequate washing of produce with water containing a disinfectant would be partially effective in reducing the FBDB. Indeed, previous studies have reported that both these interventions, i.e., prevention of cross-contamination [39,40] and washing with a disinfectant solution [40,41] can only partially reduce microbiological load. On the other hand, the correct implementation of freezing, cooking, adequate storage and processing or adequate cooling could have a higher impact to reduce FBDB. For instance, Pouillot et al. [42] showed that decreasing the shelf-life of cold smoked salmon or maintaining the average refrigerator temperature at 4 °C reduced the number of listeriosis cases by more than 75%. Regarding cooking, Smith et al. [43] showed that cooking ground beef to an internal temperature of at least 71 °C decreases the average probability of *E. coli* O157:H7 infection in Canada by a factor of 10^5^.

Beyond food safety crises, public health interventions aim to modify the behavior of food handlers. Educational food safety interventions focus on small school or community samples [44]. Among all intervention techniques, health information campaigns or mass media campaigns are able to target the consumers’ general population. The impact of health campaigns on population health has been estimated in other aspects of public health and safety (nutrition, cancer screening, road safety, and blood donation). According to published meta-analysis [45,46], the average health campaign (excluding campaigns that include legal coercion) changes the behavior of the target population according to a small effect in the short-term (*r* = 5%) which varied by the target behavior and context. To increase the effectiveness of the communication, it has been suggested that a communication strategy should combine different means of disseminating information (media, including social media, medical staff, consumer associations, etc.), address both the individual and their environment, and refer to a theoretically and empirically sound behavioral model [47].

## 5. Conclusions

This study aimed at estimating the role of specific foods as pathways contributing to foodborne disease burden and associated food-handling practices for 26 pathogen hazards. Since no quantitative data were available to perform the attribution process, expert opinion was used leading to potential under- or overestimation of the implication of a particular food or practice. Nevertheless, the main contributing food categories and practices could be identified with reasonable confidence. These estimates constitute valuable information to objectively develop intervention strategies in order to reduce the foodborne disease burden. To be most effective in reducing this FBDB, it is important to promote food safety messages to food handlers addressing the practices with the biggest impact in relation to FBDB i.e., cross-contamination, inadequate cooking and storage. These estimates are also useful to compare the potential impact of educational programs with interventions focusing on the food production chain, which could be a more effective risk reduction strategy.

## Figures and Tables

**Table 1:** Incidence of foodborne illnesses in France (circa 2010): estimated incidence, mean incidence rate of cases per 100,000 persons and attributed scores.

Hazards		Incidence	Incidence Rate	References	Scores
Bacteria, toxins and metabolites	*Bacillus cereus*	69,000	110	Van Cauteren et al. [1]	5
	*Brucella* spp.	24	0.04	Mailles et al. [22]	1
	*Campylobacter* spp.	390,000	600	Van Cauteren et al. [1]	5
	*Clostridium botulinum*	21	0.03	Van Cauteren et al. [1]	1
	*Clostridium perfringens*	120,000	180	Van Cauteren et al. [1]	5
	Histamine	167	0.3	Santé Publique France [23]	2
	*Listeria monocytogenes*	400	0.6	Van Cauteren et al. [1]	2
	*Salmonella* non-typhoidal	180,000	280	Van Cauteren et al. [1]	5
	Shiga toxin-producing *E. coli (STEC)*	18,000	28	Van Cauteren et al. [1]	4
	*Shigella* spp.	3400	5.2	Van Cauteren et al. [1]	3
	*Staphylococcus aureus*	73,000	110	Van Cauteren et al. [1]	5
	*Vibrio parahaemolyticus*	5	0.008	NRC Vibrio [24]	0
	*Yersinia enterocolitica*	21,000	32	Van Cauteren et al. [1]	4
Viruses	Hepatitis A virus	2600	4.0	Van Cauteren et al. [1]	3
	Hepatitis E virus	2300	3.5	NRC HEV [25]	3
	Norovirus	520,000	800	Van Cauteren et al. [1]	5
Parasites	*Anisakis* spp.	8	0.01	Van Cauteren et al. [15]	1
	*Cryptosporidium* spp.	101	0.2	NRC Cryptosporidium [26]	2
	*Cyclospora cayetanensis*	8	0.01	Medical network Anofel [26]	1
	*Echinococcus multilocularis*	29	0.04	Van Cauteren et al. [15]	1
	*Fasciola hepatica*	5	0.008	Van Cauteren et al. [15]	0
	*Giardia* spp.	482	0.7	Medical network Anofel [26]	2
	*Taenia saginata*	33,000	51	Van Cauteren et al. [1]	4
	*Toxoplasma gondii*, acquired	11,500	18	Van Cauteren et al. [1]	4
	*Toxoplasma gondii*, congenital	300	0.5	Van Cauteren et al. [1]	2
	*Trichinella* spp.	11	0.02	Van Cauteren [27]	1

**Table 2:** Severity scores of foodborne illnesses (based on median foodborne disability-adjusted life years (DALYs) per 1000 cases of illness) and estimated foodborne disease burden in France (circa 2010).

Hazards		Severity			Incidence	Foodborne Disease Burden (FBDB)	
		The Netherlands, 2009 [17]	EUR A, 2010 [18,19]	This Study			
		DALYs	DALYs	Score	Score	Score	% of Total FBDB
Bacteria, toxins and metabolites	*Bacillus cereus*	2.3	-	1	5	6	3%
	*Brucella* spp.	-	300	3	1	4	0.03%
	*Campylobacter* spp.	41	20	2	5	7	32%
	*Clostridium botulinum*	-	-	3	1	4	0.03%
	*Clostridium perfringens*	3.2	-	1	5	6	3%
	Histamine	-	-	1	2	3	0.003%
	*Listeria monocytogenes*	1450	8000	4	2	6	3%
	*Salmonella*, non-typhoidal	49	70	2	5	7	32%
	Shiga toxin-producing *E. coli*	143 ^1^	20	2	4	6	3%
	*Shigella* spp.	-	70	2	3	5	0.3%
	*Staphylococcus aureus*	2.6	-	1	5	6	3%
	*Vibrio parahaemolyticus*	-	-	1	0	1	0.00003%
	*Yersinia enterocolitica*	-	-	2	4	6	3%
Virus	Hepatitis A virus	167	100	3	3	6	3%
	Hepatitis E virus	460	-	3	3	6	3%
	Norovirus	2.4	2	1	5	6	3%
Parasites	*Anisakis* spp.	-	-	1	1	2	0.0003%
	*Cryptosporidium* spp.	2.9	8	1	2	3	0.003%
	*Cyclospora cayentanensis*	-	-	1	1	2	0.0003%
	*Echinococcus multilocularis*	-	2000	4	1	5	0.3%
	*Fasciola hepatica*	-	9000	2	0	2	0.0003%
	*Giardia* spp.	2.1	1	1	2	3	0.003%
	*Taenia saginata*	-	-	1	4	5	0.3%
	*Toxoplasma gondii*, acquired	3170	60	2	4	6	3%
	*Toxoplasma gondii*, congenital	6360	6300	4	2	6	3%
	*Trichinella* spp.	-	100	2	1	3	0.003%

^1^ DALY for *E. coli* O157:H7.

**Table 3:** Foodborne disease burden (90% uncertainty intervals) for exposure routes and food-handling practices implicated in the transmission of biological foodborne hazards.

Food Categories	Sub-Categories	Specific Foods	Hazards	Default in Handling Practices Contributing to the Onset of Foodborne Illnesses						No Contribution from Food Handlers at the Final Preparation
				Cross Contamination	Inadequate Washing and Disinfection of Produce	Inadequate Processing or Cooling	Inadequate Freezing	Inadequate Cooking (Including Reheating)	Inadequate Storage (Temperature and/or Shelf-Life)	
Meats	Beef	Cooked ground beef	STEC	-	-	-	-	(0.2–1.8)	-	-
			*Salmonella*	-	-	-	-	(0.2–6.6)	(0.2–6.6)	-
		Raw ground beef	STEC	-	-	-	-	-		(0.2–1.8)
			Salmonella	-	-	-	-	-	(0.2–6.6)	(0.2–6.6)
		Beef meat	*T. saginata*		-	-	(0.0–0.3)	(0.0–0.3)	-	-
	Poultry	Poultry meat	*Campylobacter* spp.	(3–29)	-	-	-	(3–29)	-	-
			*Salmonella*	(0.1–4.7)	-	-	-	(0.1–4.7)	(0.1–4.7)	-
	Pork	Pork meat	*Salmonella*	(0.1–4.7)	-	-	-	(0.1–4.7)	(0.1–4.7)	-
			*Y. enterocolitica*	(0.2–2.5)	-	-	-	(0.2–2.5)	(0.2–2.5)	-
		Raw pig liver products, boar offal	Hepatitis E virus	-	-	-	-	3.2	-	-
		Open-air pig, boar, game	*Trichinella* spp.	-	-	-	(0.000–0.003)	(0.000–0.003)	-	-
		Cooked pork meats	*L. monocytogenes*	-	-	-	-	-	(0.0–0.9)	(0.0–0.9)
			*S. aureus*	-	-	-	-	-	(0.2–2.5)	-
		Home-made cooked and salt pork meat	*C. botulinum*	-	-	(0.00–0.02)	-	-	(0.00–0.02)	-
	Other meats	Lamb, open-air pigs, imported horses	*T. gondii,* congenital	-	-	-	(0.2–2.1)	(0.2–2.1)	-	-
			*T. gondii,* acquired	-	-	-	(0.2–2.1)	(0.2–2.1)	-	-
Dairy	Unpasteurised milk	Heated milk	*Brucella* spp.	-	-	-	-	(0.00–0.03)	-	-
			STEC	-	-	-	-	(0.2–1.8)	-	-
		Raw milk	*Brucella* spp.	-	-	-	-	-	-	(0.00–0.03)
			STEC	-	-	-	-	-	-	(0.2–1.8)
	Raw milk cheeses	Fresh non-ripened cheeses	*Brucella* spp.	-	-	-	-	-	-	(0.00–0.03)
		Hard non-cooked cheeses	*Salmonella*	-	-	-	-	-	-	(1.6–12.5)
		Soft cheeses	STEC	-	-	-	-	-	-	(0.2–1.8)
			*Salmonella*	-	-	-	-	-	-	(1.6–12.5)
			*S. aureus*	-	-	-	-	-	-	(0.2–2.5)
			*L. monocytogenes*	-	-	-	-	-	(0.0–0.9)	(0.0–0.9)
	Pasteurized milk cheeses	Soft cheeses	*L. monocytogenes*	-	-	-	-	-	(0.0–0.9)	(0.0–0.9)
Eggs	-	Eggs	*Salmonella*	-	-	-	-	(1.6–12.5)	-	-
		Raw eggs products	*Salmonella*	-	-	-	-	-	(0.2–6.8)	(0.2–6.8)
Seafood	Fish	Cooked fish	*Anisakis* spp.	-	-	(0–0.0002)	(0–0.0002)	(0–0.0002)	-	-
		Raw fish	*Anisakis* spp.	-	-	(0–0.0002)	(0–0.0002)	-	-	-
		Cold smoked fish	*L. monocytogenes*	-	-	-	-	-	(0.0–0.9)	(0.0–0.9)
		Fish species with a high amount of histidine (particularly tuna)	Histamine	-	-	-	-	-	-	0.03
	Shellfish	Crustaceans	*V. parahaemolyticus*	-	-	-	-	(0–0.00003)	-	-
			*L. monocytogenes*	-	-	-	-	-	(0.0–0.9)	(0.0–0.9)
		Cooked bivalve mollusks	*V. parahaemolyticus*	-	-	-	-	(0–0.00003)	-	-
			Norovirus	-	-	-	-	(0.2–2.0)	-	-
			Hepatitis A virus	-	-	-	-	(0.2–2.0)	-	-
		Raw bivalve mollusks	*V. parahaemolyticus*	-	-	-	-	-	(0–0.00003)	(0–0.00003)
			Norovirus	-	-	-	-	-	-	(0.2–2.0)
			Hepatitis A virus	-	-	-	-	-	-	(0.2–2.0)
Raw produce	-	Frozen raw produce (red fruits, vegetables)	Norovirus	-	-	-	-	-	-	(0.2–2.0)
			Hepatitis A virus	-	-	-	-	-	-	(0.2–2.0)
		Not frozen raw produce	STEC	-	(0.2–1.8)	-	-	-	-	
			*L. monocytogenes*	-	(0.0–0.6)	-	-	-	(0.0–0.6)	(0.0–0.6)
			*Salmonella*	-	(1.6–12.5)	-	-	-	-	
			Norovirus	-	-	-	-	-	-	(0.2–2.0)
			Hepatitis A virus	-	-	-	-	-	-	(0.2–2.0)
			*Cryptosporidium* spp.	-	0.003	-	-	-	-	
			*C. cayetanensis*	-	-	-	-	-	-	0.0003
			*Giardia* spp.	-	0.003	-	-	-	-	
			*T. gondii,* congenital	-	-	-	-	-	-	(0.3–2.9)
			*T. gondii,* acquired	-	-	-	-	-	-	(0.3–2.9)
		Wild raw produce (watercress, dandelion)	*F. hepatica*	-	-	-	-	-	-	0.0003
		Red fruits and berries	*E. multilocularis*	-	-	-	-	(0.0–0.3)	-	(0.0–0.3)
Ready-made meals and complex foods	Refrigerated and processed foods of extended durability (REPFED)	All kinds of packaging	*B. cereus*	-	-	-	-	-	(0.3–2.9)	-
		Vacuum-packed	*C. botulinum*	-	-	-	-	(0.00–0.02)	(0.00–0.02)	-
	Home-made meal	Particularly those containing grain (pasta, rice, semolina) or dehydrated ingredients	*B. cereus*	-	-	(0.0–1.6)	-	(0.0–1.6)	(0.0–1.6)	-
		Particularly meat cooked in a sauce	*C. perfringens*	-	-	(0.2–2.5)	-	(0.2–2.5)	(0.2–2.5)	-
	Composite foods	Ready-made meals, cakes, extensively manipulated foods (sandwiches)	*L. monocytogenes*	-	-	-	-	-	(0.0–0.9)	(0.0–0.9)
			*S. aureus*	-	-	(0.1–1.6)	-	-	(0.1–1.6)	-
			*Shigella* spp.	0.2 (0.0–0.3)	-	-	-	-	-	(0.0–0.3)
			Norovirus	0.6 (0.2–2.0)	-	-	-	-	-	-
			Hepatitis A virus	0.6 (0.2–2.0)	-	-	-	-	-	-
		Home-made canned food	*C. botulinum*	-	-	(0.00–0.02)	-	(0.00–0.02)	-	-

**Table 4:** Attribution of foodborne disease burden (FBDB) to foods.

Food Categories	CI90 ^1^ (%)	Sub-Categories	CI90 (%)	Specific Foods	CI90 (%)
Meats	(50–69)	Beef	(4–20)	Cooked ground beef	(2–13)
				Raw ground beef	(2–13)
				Beef meat	0.3
		Poultry	(34–44)	Poultry meat	(34–44)
		Pork	(9–21)	Pork meat	(5–16)
				Raw pig liver products, boar offal	3
				Open-air pig, boar, game	0.003
				Cooked pork meats	(0–3)
				Home-made cooked and salt pork meat	(0–0.03)
		Other meats	(1–5)	Lamb, open-air pigs, imported horses	(1–5)
Dairy	(5–22)	Unpasteurised milk	(0–2)	Heated milk	(0–2)
				Raw milk	(0–2)
		Raw milk cheeses	(4–21)	Fresh non-ripened cheeses	(0–0.03)
				Hard non-cooked cheeses	(2–12)
				Soft cheeses	(2–15)
		Pasteurized milk cheeses	(0.2–1.6)	Soft cheeses	(0.2–1.6)
Eggs	(3–19)	-	-	Eggs	(2–13)
				Raw eggs products	(2–13)
Seafood	(1–6)	Fish	(0.2–1.6)	Cooked fish	(0–0.0003)
				Raw fish	(0–0.0003)
				Cold smoked fish	(0.2–1.6)
				Fish species with a high amount of histidine (particularly tuna)	0.003
		Shellfish	(1–5)	Crustaceans	(0.2–1.6)
				Cooked bivalve mollusks	(0–3)
				Raw bivalve mollusks	(0–3)
Raw produce	(6–20)	-	-	Frozen raw produce (red fruits, vegetables)	(0–3)
				Not frozen raw produce	(5–18)
				Wild raw produce (watercress, dandelion)	0.0003
				Red fruits and berries	0.3
Ready-made meals and complex foods	(8–12)	Refrigerated and processed foods of extended durability (REPFED)	(0–3)	All kinds of packaging	(0–3)
				Vacuum-packed	(0–0.02)
		Home-made meal	(3–6)	Particularly those containing grain (pasta, rice, semolina) or dehydrated ingredients	(0–3)
				Particularly meat cooked in a sauce	3
		Composite foods	(1–5)	Ready-made meals, cakes, extensively manipulated foods (sandwiches)	(1–5)
				Home-made canned food	(0–0.03)

^1^ CI90, 90% uncertainty intervals.

**Table 5:** Attribution of foodborne disease burden (FBDB) to food-handling practices.

Food Categories	CI90 ^1^ (%)	Poor Handling Practices Contributing to the Onset of Foodborne Illnesses						No Contribution from Food Handlers at the Final Preparation
		Cross-Contamination	Inadequate Washing and Disinfection of Produce	Inadequate Processing or Cooling	Inadequate Freezing	Inadequate Cooking (Including Reheating)	Inadequate Storage (Temperature and/or Shelf-Life)	
Meats	(50–69)	(6–33)	0	(0.00–0.02)	(1–3)	(13–41)	(3–15)	(1–7)
Dairy	(5–22)	0	0	0	0	(0–2)	(0.1–1.4)	(5–21)
Eggs	(3–19)	0	0	0	0	(2–13)	(0–7)	(0–7)
Seafoods	(1–6)	0	0	(0–0.0002)	(0–0.0002)	(0–3)	(0.1–1.4)	(1–3)
Raw produce	(6–20)	0	(2–13)	0	0	0	(0.0–0.6)	(3–9)
Ready-made meals and complex foods	(8–12)	(0–3)	0	(1–4)	0	(0–3)	(2–6)	(0.1–1.0)
**Total**	**-**	**(7–34)**	**(2–13)**	**(1–4)**	**(1–3)**	**(19–49)**	**(9–23)**	**(15–33)**

^1^ CI90, 90% uncertainty intervals.

## Appendix A. Source Attribution (Foods and Practices) for Foodborne Hazards in France

***Bacillus cereus:****Bacillus cereus* outbreaks are mainly associated with the consumption of refrigerated and processed foods of extended durability (REPFED) or composite meals containing grains. These estimations are in accordance with previous estimates [48,49,50]. Outbreaks generally occur with inadequate storage conditions (cold chain disruption or exceeded shelf-life) or when preparation conditions are not well controlled (cooling and cooking) for home-made meals [51].

***Brucella* spp.**: In enzootic areas, the foodborne transmission of *Brucella* spp. is linked to the consumption of contaminated unpasteurized milk or fresh non-ripened cheeses made with raw milk. Bacterial infection occurs when milk is inadequately heated before consumption but no GHPs exist when consuming raw milk or raw milk cheeses. The only intervention available at the preparation step is to avoid the consumption of this kind of product [52].

***Campylobacter* spp.**: The main considered source of foodborne campylobacteriosis was poultry meat. International source attribution studies also associate *Campylobacter* infections with poultry [53,54,55]. Although many foods can serve as vehicle for *Campylobacter* since cross contamination can occur during the food preparation, poultry meat was only considered as the ultimate source of contamination. Factors favoring the occurrence of campylobacteriosis are cross contamination but also inadequate cooking of poultry meat.

***Clostridium botulinum***: Botulism is generally associated with the consumption of home-made pork meats and canned foods inadequately prepared, stored or cooked. Vacuum-packed REPFED inadequately stored and cooked were also associated with several cases of botulism [56,57]. A few cases of infant botulism were attributed to honey consumption [58].

***Clostridium perfringens***: Outbreaks involving *C. perfringens* are generally attributed to home-made meals, particularly beef meat based products [32,48,49,50]. These outbreaks require an error during the preparation (inadequate cooling or reheating) or the storage (inadequate temperature) steps.

**Shiga toxin-producing *E. coli* (STEC)**: Foods associated with STEC infections are ground beef, raw milk and soft cheeses made with raw milk, and produce eaten raw. The importance of beef and produce is consistent with previous estimations [32,49,59]. Ground beef is incriminated when it is eaten raw (tartare) or insufficiently cooked. The only interventions at the preparation step for dairy products is to heat raw milk or to avoid eating raw milk products, particularly for young children who demonstrate a higher host susceptibility. The contamination of produce can be reduced by applying washing and disinfection procedures.

***Listeria monocytogenes***: Listeriosis cases are related to the consumption of a large variety of foods. Most often implicated foods are ready-to-eat foods supporting the growth of the pathogen during the shelf-life. The major group of foods considered were cooked pork meats, soft cheese made with raw or pasteurized milk, cold smoked fish, crustaceans, raw vegetables and composite foods. Dairy products and deli meats were also regarded as major vehicles by Batz, Hoffmann [32]. Produce and seafood were considered as secondary sources [32]. Disruption of the cold chain or exceeded shelf-life during storage are, then, contributing factors but listeriosis can also occur without food handlers being at fault when consumers exhibit a high host susceptibility.

***Salmonella enterica*, non-typhoidal**: Salmonellosis can result from the consumption of numerous foods. The main considered foods were ground beef, chicken and pork meats, raw milk cheeses, raw eggs and egg products, raw produce. These implicated foods are consistent with published estimations ([32,48,49,59]. The favoring food-handling conditions (food eaten raw or insufficiently cooked) and intervention strategies (cooking, washing and disinfection, removal) are the same as with STEC for meat products, cheeses and produce. By contrast with STEC, inadequate storage was also considered as a significant favorable factor for salmonellosis associated with meats. Infections implicating eggs are related to insufficient cooking and those linked to raw egg products are sometimes associated with cross contamination and inadequate storage temperature.

***Shigella***: Foodborne shigellosis is mainly associated with meals prepared and mishandled in food-service establishments [60]. The contamination can be due to cross contamination with the food handlers during the preparation or prior to food preparation and no intervention strategy is then available at the preparation step.

***Staphylococcus aureus****: S. aureus* toxin is mainly associated with raw milk soft cheeses and numerous composite dishes [48,49,50]. No interventions strategies are available for food handlers when cheeses are contaminated. On the other hand, outbreaks linked to composite dishes are generally related to inadequate temperature control during the preparation or the storage.

***Vibrio parahaemolyticus***: Foodborne infections involving *V. parahaemolyticus* are linked to shellfish eaten raw or insufficiently cooked. For raw bivalve mollusks, an inadequate storage temperature increases the probability of foodborne infection [61].

***Yersinia enterocolitica****:* In France, *Y. enterocolitica* infections are mainly associated with pork meat [62]. Adequate storage conditions and a sufficient cooking are regarded as effective GHPs applicable by food handlers.

**Histamine**: Histamine is mainly produced in fish species with a high amount of histidine, particularly tuna from the *Scombridae* family. No intervention is available for food handlers at the preparation step since histamine is produced immediately after fishing.

**Norovirus, hepatitis A virus**: Foodborne transmission of norovirus and hepatitis A virus involves bivalve mollusks, raw produce (frozen or not) and extensively manipulated meals like sandwiches. These foods are the main categories implicated in outbreaks [49]. Hoffmann et al. [59] and Batz et al. [32] identified produce and seafood as major foods for the transmission of norovirus in the USA. No intervention strategies are available for food handlers when these foods are eaten raw. Adequately cooking bivalve mollusks is effective to destroy the viral particles. As for manipulated complex foods, the contamination generally occurs during food handling by cross contamination.

**Hepatitis E virus**: Foodborne transmission of hepatitis E virus is linked to the consumption of pig liver products or food containing boar offal insufficiently cooked [63]. Seafood, fruit and vegetables were not considered as significant routes [48].

***Anisakis* spp**.: *Anisakis* spp. are parasites found in infected fishes. A rapid evisceration of fishes is considered a good preparation practice and is recommended to avoid the transit of larvae from abdominal cavity to muscles. Parasites can be destroyed by adequate freezing or cooking which constitute relevant intervention strategies at the preparation step [64].

***Cryptosporidium* spp., *Cyclospora cayetanensis, Giardia* spp**.: These protozoan parasites are mainly associated with the consumption of raw produce. Contrary to Havelaar et al. [48], meats and shellfishes were considered as minor sources for *Cryptosporidium* and *Giardia*. But this was consistent with the estimations of Hoffman et al. [59] and Batz et al. [32] who also identified produce as the major source of contamination for *Cryptosporidium* and *Cyclospora*. Except for *Cyclospora* which is particularly resistant [65], the adequate washing and disinfection of produce are useful intervention strategies applicable at the food-handling step to reduce the probability of infection.

***Echinococcus multilocularis***: The ingestion of *E. multilocularis* eggs is associated with the consumption of contaminated red fruits and berries or vegetables harvested near the ground. Eggs of the parasite are not eliminated at the food-handling step by washing and disinfection, and they are not completely inactivated by freezing [66]. Adequate cooking can destroy eggs of this parasite.

***Fasciola hepatica****:* Fasciolosis is contracted by eating wild raw produce like watercress and dandelion. Parasitic *metacercariae* encysted on plants are highly resistant to washing and disinfection [67] then no intervention strategies are usable by final food handlers.

***Taenia saginata****: T. saginata* is a parasite exclusively transmitted by beef meat. Adequate freezing or cooking are effective GHPs to destroy *Cysticercus bovis* localized in meat [68].

***Toxoplasma gondii****: Toxoplasma* oocysts are transmitted when eating contaminated raw vegetables. Wahsing and disinfection of vegetables is not effective to reduce the oocysts load present on produce [69]. Cysts are present in tissue of infected animals. Eating lamb meat, meat from open-air pigs or horse meat is generally recognized as the source of toxoplasmosis when insufficiently cooked. Pork, beef and game meats were estimated as major routes in the USA [32,59]. Cysts can be destroyed by freezing or adequate cooking of meats.

***Trichinella* spp**.: Human trichinellosis is associated with the consumption of infected meats from open-air pigs, boars or game meats. Intervention strategies at the food-handling step consisting in freezing or cooking can effectively destroy the encysted larvae [70].
